# Minimum Time Dose in Nature to Positively Impact the Mental Health of College-Aged Students, and How to Measure It: A Scoping Review

**DOI:** 10.3389/fpsyg.2019.02942

**Published:** 2020-01-14

**Authors:** Genevive R. Meredith, Donald A. Rakow, Erin R. B. Eldermire, Cecelia G. Madsen, Steven P. Shelley, Naomi A. Sachs

**Affiliations:** ^1^Master of Public Health Program, Cornell University, Ithaca, NY, United States; ^2^Department of Population Medicine and Diagnostic Sciences, Cornell University, Ithaca, NY, United States; ^3^Atkinson Center for a Sustainable Future, Cornell University, Ithaca, NY, United States; ^4^Section of Horticulture, School of Integrative Plant Sciences, Cornell University, Ithaca, NY, United States; ^5^Flower-Sprecher Veterinary Library, Cornell University, Ithaca, NY, United States; ^6^Department of Plant Science and Landscape Architecture, University of Maryland, College Park, MD, United States; ^7^Therapeutic Landscapes Network, Washington, DC, United States

**Keywords:** nature, mental health, well-being, stress, time dose, university, college

## Abstract

**Background:** Across the U.S., college and university students exhibit high levels of stress, anxiety, depression, and other mental health issues. While counseling, medications and, in more severe cases, hospitalization are all appropriate treatments for such conditions, an increasing body of evidence has demonstrated that spending time in nature can provide tangible benefits for mental health and well-being. The aim of this study was to define a “dose” of time in nature that could be prescribed to college-age students, as a preventative and supportive mental health and well-being intervention. The specific objectives of this scoping review were thus: to define the minimum amount of time in nature that results in positive impact on mental health and well-being for college-aged students; to describe the types of engagement with nature that elicited the impact; and to describe and explore the most commonly used measure of effect pre- and post-time in nature.

**Methods:** This scoping review was conducted following the PRISMA-ScR Checklist. A review protocol was developed but not registered. Fourteen bibliographic databases were searched and all results were blindly screened using established inclusion criteria. All titles and abstracts were screened by at least two reviewers, a third being used as a tie-breaker if needed. Studies were included if: subjects were of average college age; they examined a treatment of time (hours or minutes) in nature; they examined change in measures of mental health and well-being pre- and post-exposure; they compared participants across at least two environments; the study was published in English or French; and if the study was <20 years old.

**Results:** Initially, 11,799 titles were identified and once de-duplicated, 10,917 titles were screened. One hundred fifty-five papers were given full text reviews, of which 14 studies were included in this review. In summary, 13 of the 14 papers explicitly noted that the participants were college students. Two-thirds of the studies (*n* = 10) took place in Japan. One study took place in Sweden, and the remaining studies took place in the United States (*n* = 3). These studies show that, when contrasted with equal durations spent in urbanized settings, as little as 10 min of sitting or walking in a diverse array of natural settings significantly and positively impacted defined psychological and physiological markers of mental well-being for college-aged individuals. Within the included studies, 22 different measures were used to assess the effects of nature doses on mental health and well-being.

**Conclusions:** This review provides time-dose and activity-type evidence for programs looking to use time in nature as a preventative measure for stress and mental health strain, and also demonstrates opportunities in six specific foci for more research in this area.

## Introduction

### Rationale

While the U.S. continues to hold the top ranking among university systems worldwide, American college and university students are experiencing unprecedented levels of stress, depression, and other psychologically-debilitating conditions (Williams and Leahy, 2018). The causes of this collective emotional distress are many, from competition for grades, to technologically-prompted isolation, to severe financial pressures (Eagan et al., 2015).

There is ample evidence of the current public health crisis of student well-being on American campuses. Within the 12 months prior to a 2017 survey, responding college students reported more than average or tremendous stress (67%); feeling overwhelming anxiety (61%); hopelessness (51%); and 13.2% had been diagnosed or treated for depression or anxiety (American College Health Association, 2017). These high levels of mental health strain are reflective of the general population, in which one fifth of the global population experienced mental illness sometime in the previous 12 months (World Health Organization, 2017).

Public health and healthcare staff and administrators at colleges and universities in the U.S. are aware of these challenges and recognize that their institutions' educational missions cannot be achieved without attending to the mental and behavioral health concerns of their students. While they are taking steps to address these needs, counseling and intervention demands can be overwhelming (Association of University and College Counseling Center Directors Annual Survey, 2017; USA Today, 2017). A recent study of 112 presidents and student affairs leaders at 2- and 4-year post-secondary education institutions found that mental health issues among enrolled students was the respondents' number one health concern (The Chronicle of Higher Education, 2017). In 2014, 94% of Counseling and Psychological Services (CAPS) center directors indicated that the number of students with severe psychological problems continues to increase on their campuses, yet almost two-thirds of students who meet the criteria for depression do not get help, and only about four percent of students with a history of alcohol use disorder receive services of any kind (Douce and Keeling, 2014). In fact, 76% of college counseling directors reported that they had to reduce the number of visits for non-crisis patients to cope with the increasing overall number of clients (Gallagher, 2015).

While the most debilitating cases of student psychological problems, such as suicidal ideation, self-laceration, and severe substance abuse, must be addressed through comprehensive approaches including counseling, prescription medication, and possible hospitalization, multiple studies have demonstrated the beneficial upstream preventative effects of spending time in natural settings on emotional well-being and cognitive acuity (Maller et al., 2006; Van den Berg et al., 2007; Bratman et al., 2012; Cox et al., 2017; Hansen et al., 2017; Antonelli et al., 2019). And, while four recent review articles summarize and reinforce the mental health and well-being benefits from time in nature (James et al., 2016; Crouse et al., 2017; Hansen et al., 2017; Antonelli et al., 2019), there is no clear summary of how much time is needed to elicit these positive results, particularly among college-age students. Thus, as public health practitioners and educators with interest in supporting student mental health and well-being, we sought to examine the available evidence to make informed decisions related to campus-based interventions in the U.S. This included identifying what accessible and sustainable dose of time in nature is required to elicit a positive impact on the mental health and well-being among people of college-age, and to describe what types of engagement with nature (i.e., passive vs. active engagement) provide said impacts.

### Theoretical Basis

A variety of theories of how nature impacts human health have been advanced over the past 150 years. In 1865, Olmsted reflected on the role of natural scenery in psychological restoration: it “employs the mind without fatigue and yet exercises it; tranquilizes it and yet enlivens it; and thus, through the influence of the mind over the body, gives the effect of refreshing rest and reinvigoration to the whole system” (quoted in: Nash, 2014).

In recent decades, several theories have been proposed to explain the mechanism by which time spent in nature promotes an improved psychological state. *Attention-restoration theory (ART)* developed by Kaplan and Kaplan in the 1980s, postulates that prolonged use of directed (voluntary) attention, as demanded by the complex and technologically-driven modern world, causes mental fatigue and associated loss of focus and increased irritability. Experiences in the natural world, according to this theory's proponents, promote a restorative environment which allows the brain's directed attention to rest and recover from the rigors of problem solving (Kaplan, 1995).

In contrast to ART, *stress-reduction theory*, developed by Ulrich et al. in the 1980s, argues that natural environments facilitate reductions in physiological arousal following stress, rather than the restoration of directed attention (Ulrich et al., 1991). This theory posits that, in response to external stressors, shifts occur in the body's cardiovascular, skeletomuscular and neuroendocrine systems. Time spent in natural settings, or even viewing natural scenes through a window or on a screen, can result in positive changes in physiological activity levels and then lead to a more positively-toned emotional state (Hartig et al., 2014). Landscaped settings, such as those found at botanic gardens or parks, have also been shown to have a role in stress reduction (Kohlleppel et al., 2002; Grahn and Stigsdotter, 2003). These findings are consistent with other studies that have found that anything from a grassland to a waterfall can provide the restorative mental health benefits of nature (Van den Berg, 2008; Hansen et al., 2017; Antonelli et al., 2019).

Current levels of stress and poor mental health among students at institutions of higher learning are acknowledged to be unacceptably high, and time spent in nature has been shown to offer relief from stress, depression, and lack of focus (Lau and Yang, 2009). The particular natural experience an individual seeks can vary from a seemingly untouched forest to a fully designed landscape. Much research has indicated that it is the time spent in nature, not the “nature of the nature,” that is most critical (Wolf and Robbins, 2015; Bratman et al., 2019).

A few recent studies have focused specifically on this question of appropriate time dose in nature. One study examined the effect of nature experiences on reductions in salivary cortisol and alpha-amylase concentrations, two biomarkers of physiological stress. These researchers found that a nature duration between 20 and 30 min, three times per week, was most efficient (Hunter et al., 2019). A second study looked at weekly time in nature, comparing individuals who spent various intervals to those who spent no time in nature. These researchers found that spending at least 120 min in nature per week led to significantly higher self-reports of positive health and well-being (White et al., 2019). While neither of these studies focused on college-aged students, they provide evidence of the feasibility of prescribing specific time doses in nature.

### Objectives

The objectives of this study were to comprehensively scan the available literature to (1) identify what *accessible and sustainable dose of time in nature* appears to elicit a positive impact on *mental health* in people of college-age, (2) describe what types of engagement with nature (i.e., passive vs. active engagement) provide said impacts, (3) describe the most commonly used measures of effect, and (4) identify the strengths and gaps in the literature (methods and understanding) to guide future research.

Accessible and sustainable doses of time in nature were defined as minutes and hours in types of natural areas that could be easily and routinely reached from a college campus by any student, regardless of means; this excluded, for example, multi-day outdoor retreats or experiences in “exotic” places such as deep wilderness, mountain tops, etc. Mental health was defined as psychological elements linked to general well-being (e.g., low stress, lack of depression, happiness, ability to focus). While the term “nature” may strictly be defined as “all the animals and plants in the world and all the features, forces, and processes that exist or happen independently of people, such as the weather, the sea, mountains, reproduction, and growth” (The Cambridge Dictionary Online)^1^, in practice, much of the research views nature more expansively as including elements of the built or designed environment. For the purpose of this review, nature was defined as green spaces, including manicured urban parks, urban woods, and relatively undisturbed natural sites.

### Research Question

This study looked to define how much time, doing what activities in nature, has a positive impact on mental health among college-age students, and what methods can be used to measure effect.

## Methods

### Study Design

A scoping review approach was used to guide this study, allowing the research team to identify and review available and relevant literature, to surface and describe themes and gaps, as a step toward more focused research.

### Participants, Interventions, Comparators

Subjects of interest for the review included people of average college age (no younger than 15, no older than 30), and interventions of interest for the review included a defined/measured period of time in nature engaged in a defined activity. Measures of interest for the review included changes in biological and/or self-reported measures linked to mental health and well-being.

### Scoping Review Protocol

This scoping review was conducted following the PRISMA (preferred reporting for items for systematic reviews and meta-analyses, http://www.prisma-statement.org/) checklist for standards for systematic reviews. Part-way through the implementation of this scoping review, the PRISMA-ScR Checklist was published, and this scoping review has been adapted to follow these newly published guidelines (Tricco et al., 2018). A scoping review protocol, available upon request, was developed but not registered.

### Search Strategy and Data Sources

Bibliographic databases were searched on December 15, 2016, including Web of Science (All Databases, which included BIOSIS Citation Index, BIOSIS Previews, CAB Abstracts, Current Contents Connect, Data Citation Index, Derwent Innovations Index, FSTA, KCI-Korean Journal Database, Russian Science Citation Index, SciELO Citation Index, and Zoological Record) (1864—present), PsychInfo (1597—present), ProQuest Dissertations and Theses (1861—present), and PubMed (1966—present). Searches were re-run on May 18, 2017 to capture fresh publications.

Searches in Web of Science and PsychInfo used search terms including synonyms of nature, exposure, time elements, university students and terms relative to mental health and wellness (see Appendix A for complete search details). ProQuest Dissertations and Theses, a bibliographic database that has limited search capabilities, was searched with: *su((“health”) AND “nature”)*. PubMed, a bibliographic database that indexes some literature relevant to this review, was searched using the following string: *(health[MeSH Terms]) AND nature[MeSH Terms]*. Preliminary attempts to build a search in PubMed using text words ([tw]) or title and abstract words ([tiab]) to capture entries not yet indexed by MeSH Terms yielded tens of thousands of entries that were outside the scope of this review, rendering the search result set too cumbersome to screen. Therefore, we only used MeSH searching in PubMed.

Additionally, the website Green Cities:Good Health (http://depts.washington.edu/hhwb/) was searched; experts in the field were contacted directly for updates on relevant research; and additional resources were identified via careful scrutiny of bibliographies of included literature.

### Study Selection, Data Extraction

Studies were eligible for inclusion in this scoping review if they met the following criteria: (1) subjects were of average college age (no younger than 15, no older than 30); (2) the study examined a treatment of time (hours or minutes) in nature (excluding exposure to nature in urban microenvironments, e.g., looking at nature through a window or on a screen); (3) a change, if any, in mental health status was noted; (4) studies compared participants across at least two environments; and (5) the study was published in English or French. Studies that did not meet these criteria were excluded, as were studies that tracked change in mental health status based on days and weeks immersed in nature (e.g., Outward Bound-type activities) because this is not generally accessible to all students; studies that looked at exercise in nature, because physical activity could be a confounder; studies that were more than 20 years old; and studies that compared time outdoors to time indoors.

All identified resources were managed in Rayyan (https://rayyan.qcri.org/welcome). After removing duplicate records, all titles and abstracts were screened for relevence (inclusion criteria) by at least two reviewers, a third being used as a tie-breaker if needed. For resources not excluded based on titles and abstracts, full manuscripts were reviewed by at least two reviewers. For each included study, study characteristics, sample characteristics, and results were extracted to thematic tables in Microsoft Word and Microsoft Excel.

### Data Analysis

Given that the subject matter of this scoping review extends into disciplines that rely on qualitative as well as quantitative research, and because many of the included studies relied on subjective measures and small sample sizes, this scoping review used a thematic analysis to look across studies and pull out themes for future consideration.

## Results

### Included Studies and Characteristics

**Figure 1:**
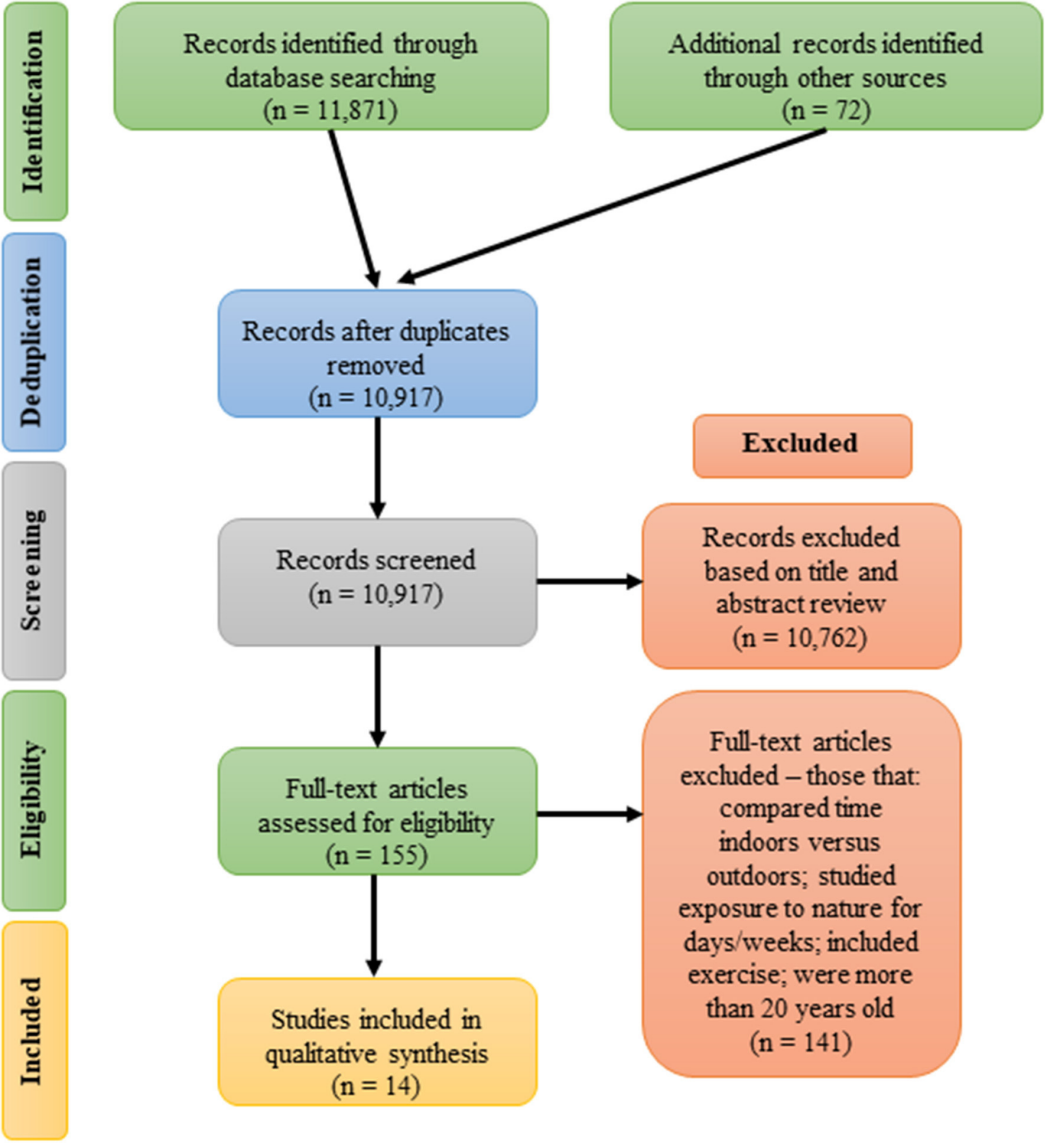
PRISMA flow diagram.

In sum, 11,799 resources were identified and once de-duplicated, 10,917 titles and abstracts were screened for relevance and inclusion; these included both gray and white literatures. Rayyan (https://rayyan.qcri.org/welcome) was used with the blind function turned on to reduce bias during screening. Some 10,762 resources were excluded for not meeting inclusion criteria based on a review of titles and abstracts. A total of 155 papers were considered via full-text review, and of those, 14 papers were included for review (Figure 1). All included papers were from peer-reviewed journals. From these included papers, data were extracted and collected to define participant age, type of exposure to nature, type of activity in nature, types of data collected, measures used, and resulting change in mental health status. This information is summarized in Table 1.

**Table 1:** Characteristics of included studies.

**Study**	**Type of study**	**Sample**	**Location**	**Dose**	**Activity**	**Type of Nature**	**Measures**
#1 Berman et al. (2008)	Within-subject crossover: natural vs. urban	38 College age Mean 22.3 years 61% female	U.S.A. Michigan	50-min, once	Walk	Secluded, tree-lined arboretum vs. urban walk on heavily-trafficked street	Affect (PANAS) Attention (backwards digit-span task)
#2 Hartig et al. (2003)	Within-subject crossover: natural vs. urban	112 College age Mean 20.8 years 50% female	U.S.A. California	50-min, once	Slow-pace walk (“saunter”)	Nature reserve with dirt road vs. urban city with sidewalks	Physiology (blood pressure) Affect (ZIPERS) (OHS) Attention (NCPCT) (memory—Smith and Miles)
#3 Johansson et al. (2011)	Within subject crossover: natural vs. urban	20 College age Age 20–29 50% female	Sweden	50-min, once	Brisk walk	Landscaped park with no roads or large buildings vs. urban walk on sidewalks adjacent to trafficked road with many buildings	Affect (EFI scale) (NMS scale) (PSS) Attention (Symbol Substitution Test)
#4 Lee et al. (2009)	Within-subject crossover: natural vs. urban	12 College students Mean 21.3 years Male	Japan	15-min, once	Sit and view	Dense forest vs. urban commercial street with traffic and dense buildings	Physiology (cortisol) (blood pressure) (heart rate) Affect (self-report)
#5 Lee et al. (2011)	Within-subject crossover: natural vs. urban	20 College students Mean 21.2 years Male	Japan	15 min, once	Sit and view	Natural area on campus with trees and pond vs. urban commercial area	Physiology (heart rate) (heart rate variability) (cortisol) Affect (SD) (POMS) (STAI) (SCL-90-R)
#6 Mayer et al. (2009)	Study #1 Urban walk, nature preserve. Not crossover	76 College age No age (intro class) 66% female	USA	10 + 5 min, once	Walk Sit	Natural setting walk vs. urban walk	Physiology (PANAS) Affect (self-awareness)
#7 Park et al. (2007)	Within-subject crossover: natural vs. urban	12 College students Mean 22.8 years Male	Japan	20 min, each	Walk + Sit	Forest area vs. urban commercial area	Physiology (neural activity) (cortisol) Affect (comfort) (calm)
#8 Park et al. (2008)	Within-subject crossover: natural vs. urban	12 College students Mean 21.3 years Male	Japan	15 min, once	Sit and view	Dense forest view vs. urban street view (dense, traffic)	Physiology (cortisol) (heart rate) (heart rate variability) Affect (self-report) (relaxation)
#9 Park et al. (2010)	Summary of 24 within-subject crossover: natural vs. urban	280 College students Mean 21.7 years Male	Japan	11–21 min, 12–16 min sit, once	Walk + Sit	Forest walk + sit vs. urban walk + sit	Physiology (cortisol) (heart rate) (blood pressure) (sympathetic nerve activity) Affect (POMS)
#10 Song et al. (2013)	Within-subject crossover: natural vs. urban	13 College age Mean 22.5 years Male	Japan	15 min, once	Walk	Urban park vs. city area	Physiology (heart rate) (heart rate variability) Affect (SD) (POMS) (STAI)
#11 Song et al. (2014)	Within-subject crossover: urban park vs. urban	17 College age Mean 21.2 years Male	Japan	15 min, once	Walk	Urban park with trees and flowers vs. city area	Physiology (heart rate) (heart rate variability) Affect (SD) (POMS) (STAI)
#12 Song et al. (2015)	Within-subject crossover: urban park vs. urban streets	23 College age Mean 22.3 years Male	Japan	15 min, once	Walk	Urban park walk (trees, pond) vs. urban street walk (residential area)	Physiology (heart rate) (heart rate variability) Affect (SD) (POMS) (STAI) (relaxation)
#13 Tsunetsugu et al. (2007)	Within-subject crossover: natural vs. urban	12 College students Mean 22.0 years Male	Japan	15 min+15 min, once	Walk Sit and view	Urban park walk (trees, pond) vs. urban street walk (residential area)	Physiology (blood pressure) (cortisol) (heart rate) (heart rate variability) (immunoglobulin A) Affect (self-report)
#14 Tsunetsugu et al. (2013)	Summary of 4 within-subject crossover: natural vs. urban	48 College age Mean 21.1 years Male	Japan	15 min, once	Sit and view	Natural setting (no street or buildings in view) vs. urban street view (dense, traffic)	Physiology (blood pressure) (heart rate) (heart rate variability) Affect (POMS) (self-report) (refreshed)

All included papers summarized studies that were implemented and published since 2000 and included people of college age; 13 of the 14 papers noted that the participants were college students. The studies had a minimum of 12 participants under investigation; four studies had 12 participants, and two studies had more than 100 participants (112 and 280 participants). A majority of studies (*n* = 10) had only male subjects; these studies were all conducted in Japan, and represent 449 people measured. The remainder of the studies had both males and females included, with more females studied than men overall (158 females, 96 males, 3 gender not reported). Two-thirds of the studies (*n* = 10) took place in Japan, led by a seemingly related study group (Park, Song, Lee, and Tsunetsugu). One study took place in Sweden, and the remaining studies took place in the United States (*n* = 3). All study sites, both natural and urban, appeared to be within short walking or driving distances from the subjects' campuses, and would therefore be accessible to most students.

### Synthesized Findings

As detailed in Table 1, all 14 studies compared participants' measured physiological and affective responses following activities undertaken across two environments: a natural setting vs. an urbanized setting. The “nature” environments used in the studies were diverse, including college natural areas (*n* = 1; study #5), forests (*n* = 4; studies #4, 7–9), nature reserves or nature areas (*n* = 4; studies #1, 2, 6, 14), and urban parks (*n* = 5; studies #3, 10–13). The “urban” environments used for comparison (*n* = 14) were all more built up, described as urban environment, city, or urban street view. The 14 studies ranged in the type of activity with which subjects were engaged in nature, including sitting (*n* = 8; studies #4–9, 13–14), walking (*n* = 10; studies #1–3, 6–7, 9–13), and walking quickly (*n* = 1; study #3) (counts not mutually exclusive).

Also as summarized in Table 1, and detailed in **Tables 3**–**5**, the “dose” of nature evaluated in the 14 studies ranged from 10 to 50 min. Ten studies (studies #4–12, 14) looked at a time dose of 10 to 21 min, and three studies (studies #1–3) looked at a time dose of 50 min. One study (study #13) considered a 30-min dose. In all cases, effect of time in nature was measured after one event or one dose.

All but one study (study #6) utilized a within-subject cross-over design whereby all participants were studied under two conditions to help rule out confounders based on sample distribution.

A total of 22 different measures were employed in the 14 studies to assess changes in participants' physiology and affect following time in nature. These assessment methods, described in Table 2, can largely be grouped into three categories.

1. *Physiology*. A person's body reacts to stress, and physiological monitoring and tests can be used to assess changes in a person's body via known markers, including heart rate, heart rate variability, blood pressure, and salivary cortisol levels. A full 93% (*n* = 13) of the studies (all but study #1) included in this review used measures to assess the participants' physiological changes due to time in nature.
2. *Affect*. Psychometric tests of affect, mood, and volition have been used for many years to assess people's disposition. All 14 of the studies included in this review used at least one measure of affect to assess the participants' psychological changes due to time in nature. As detailed in Table 2, this included 17 different assessments, 12 standardized and validated assessments (the Exercise-induced Feeling Inventory (EFI; Gauvin and Rejeski, 1993); the Negative Mood Scale (NMS; Scott et al., 2001); the Overall Happiness Scale (OHS; Campbell et al., 1976); the Positive and Negative Affect Schedule (Watson et al., 1988; Crawford and Henry, 2010; PANAS); the Profile of Mood States (POMS; Heuchert and McNair, 2019); the Perceived Stress Scale (PSS; Cohen, 1994); the Refreshed Scale (Mackay et al., 1978); the Semantic Differential Scale (SD; Summers, 1970); the Symptom Checklist-90-Revised (SCL-90-R; Derogatis, 1994); the State-Trait Anxiety Inventory (STAI; American Psychological Association, 2019a); the Zuckerman Inventory of Personal Reactions (ZIPERS; Zuckerman, 1977); the Reflection Rumination Questionnaire (RRQ; Trapnell and Campbell, 1999) and five study-specific assessments.
3. *Attention*. Three of the studies included in this review (studies #1–3) also used measures of attention to assess whether time in nature influenced participants' memory or ability to focus. Four different standardized tests were used, including: the Necker Cube^2^ Pattern Control Test (NCPCT; De Young, 2016); the Backwards digit-span task (Berman et al., 2008); the Symbol Substitution Test (SST; Johansson et al., 2011); and the Memory-Loaded Search Task (Hartig et al., 2003).

**Table 2:** Description of measures used in included studies.

**Type of test**	**Test + meaning of test result**
Physiology (American Psychological Association, 2019b)	Sympathetic Nerve Activity • Under stress, the “fight or flight” response is activated in the body by the sympathetic nervous system (SNS). The SNS acts to release cortisol into the blood. SNS activity can be measured by heart rate variability, via the R-R interval (time duration between two consecutive R waves as measured on an electrocardiogram) (Park et al., 2008). High frequency elements act as a marker of parasympathetic activity (calm); a formula using low and high frequency elements acts as a marker of sympathetic activity (stress) (Lee et al., 2011). Salivary Cortisol Levels • Cortisol is a naturally-occurring hormone that is released under stress. When cortisol increases, the body responds by increasing heart rate, blood pressure, muscle tension, and respiratory rate. Levels of cortisol can be measured via mouth, with a swab or pipette that absorbs saliva. Heart Rate • Heart rate spikes when cortisol levels increase (under stress), and can be measured manually or remotely via monitors. Blood Pressure • Blood pressure spikes when cortisol levels increase (under stress) as blood vessels dilate to get more blood to the body; this is measured via a cuff. Secretory Immunoglobulin A • A marker of stress, as measured via saliva (Fan et al., 2008; Benham et al., 2009).
Affect	EFI: Exercise-Induced Feeling Inventory (Gauvin and Rejeski, 1993) • Self-report questionnaire to assess positive and negative affect • 12 questions to assess current feelings (calm, energetic, enthusiastic, fatigued, happy, peaceful, refreshed, relaxed, revived, tired, upbeat, worn out) • All items scored on a 5-point Likert scale (“do not feel” to “feel very strongly”) NMS: Negative Mood Scale (Scott et al., 2001) • A measure of immediate and/or persistence affective responses • 19 items measured (e.g., worried, anxious, sad, angry, irritable, rushed) • All items scored on a 5-point scale (“not at all” to “very much”) OHS: Overall Happiness Scale (Campbell et al., 1976) • A measure of quality of life • All items scores on a 100-point thermometer-like graph (“very unhappy” to “very happy”) PANAS: The Positive and Negative Affect Schedule (Watson et al., 1988; Crawford and Henry, 2010) • Validated self-report questionnaire to assess positive and negative affect • Mostly used in research settings • 10 questions for positive affect (active, alert, attentive, determined, excited, enthusiastic, inspired, interested, proud, strong); 10 for negative affect (afraid, ashamed, distressed, guilty, hostile, irritable, jittery, nervous, scared, upset). • All items scored on a 5-point Likert scale (“not at all” to “extremely”) POMS: Profile of Mood States (Heuchert and McNair) • Validated self-report questionnaire to assess mood disturbance • Measures seven dimensions of fluctuating feelings and affect states [anger-hostility (AH), confusion-bewilderment (C), depression-dejection (D), fatigue-inertia (F), tension-anxiety (TA), vigor-activity (V)] • Long form has 65 questions; short form has 35 questions • All items scored on a 5-point Likert scale (“not at all” to “extremely”) PSS: Perceived Stress Scale (Cohen, 1994) • Validated self-report questionnaire to assess perceived stress • 10 items that measure feeling for stress over the last month (using descriptors such as unpredictable, uncontrollable, and overloaded) • All items scored on a 5-point scale (“never” to “very often”); higher score indicates higher perceived stress Refreshed (Mackay et al., 1978) • (It appears that this measure might come from Mackay's scale that was published in 1978. The scale uses 30 adjectives to allow a respondent to sell assess stress and arousal. It is unclear if the full scale was used, or just questions that relate to feelings of being refreshed.) SD: Semantic Differential (Summers, 1970) • Pairs of adjectives presented (i.e., comfortable–uncomfortable, soothed—aroused, natural–artificial) • Respondents plot their “position” on a scale between the two adjectives; 3-, 5-, or 7-point scale can be used SCL-90-R: Symptom Checklist-90-Revised (Derogatis, 1994) • Validated self-report tool that evaluates a range of psychological problems and symptoms of psychopathology • Can be used in research or in clinical setting; good for measuring change in symptoms, including depression and anxiety • 90 questions in 9 symptom dimensions (anxiety, depression, hostility, interpersonal sensitivity, obsessive-compulsive, paranoid ideation, phobic anxiety, psychoticism, somatization scored on a 5-point scale STAI: The State-Trait Anxiety Inventory (American Psychological Association, 2019a) • Measure of trait and state anxiety • Can be used in research (measure of distress) or in clinical setting (diagnose anxiety) • 20 items measured for trait anxiety (e.g., tension, worry, calm, secure); 20 items measured for state anxiety (e.g., worried too much, content, steady person) • All items scored on a 4-point scale (“almost never” to “almost always”) • Higher score means higher anxiety ZIPERS: Zuckerman Inventory of Personal Reactions (Zuckerman, 1977) • Validated self-report questionnaire to assess feelings and reactions • 12 measures in five domains (attentiveness, anger/aggression, fear, positive affect, sadness). • All items scored on a 5-point scale (“not at all” to “very much”) • A sensitive measure in previous experimental research on the relative restorative potentials of natural and urban environments RRQ: Reflection Rumination Questionnaire (Trapnell and Campbell, 1999) • Two scales that measure rumination and reflection • 12 items measured for rumination (e.g., “My attention is often focused on aspects of myself I wish I'd stop thinking about”) • All items scored on a 5-point scale (“strongly disagree” to “strongly agree”) • Higher means of the sum of scores indicate higher degrees of rumination. Self-Reports: (non-validated measures) • Comfort, Calm, Positive Feeling, Relaxation, Self-awareness
Attention	NCPCT: Necker Cube Pattern Control Test (De Young, 2016; Zealand) • Test of capacity to direct mental effort, the ability to inhibit one response over another. When placed under prolonged demand, the ability to direct mental focus diminishes; this decreases mental effectiveness. • Participants view a sketch of a 3-D cube, and note (press the spacebar) when they see one orientation vs. the other. After a baseline measure, participants try to control seeing one perspective vs. another, and note when the orientation shifts. A lower score (over 30 s) shows greater attention. Backwards Digit-Span Task (Berman et al., 2008) • Participants hear sequences of digits and are asked to repeat them in reverse order. • Sequences can vary in length (three to nine digits were used in noted study) • Correct sequences were scored the same, independent of sequence length • The backwards digit-span task depends on directed-attention abilities because participants must move items in and out of their attentional focus which is a major component of short-term memory. SST: Symbol Substitution Test (Johansson et al., 2011) • A symbol substitution test requires sustained directed attention. • A subject writes numbers into a series of blanks, each of which is paired with one of nine symbols. The appropriate number for a symbol is indicated by a key. • After a practice trial, the subject is given 60 s to fill in as many of the 110 available blanks as possible. The score is the number of correctly assigned numbers. Memory-Loaded Search Task (Hartig et al., 2003) • A test of attention • Subjects search lines of letters for five target letters given at the beginning of each line. • Subjects memorize the five given targets, and then search through one line of text, once, to find the targets. • Over a 10-min period, subjects are encouraged to go quickly, but to be accurate. • Task is scored on accuracy: percent of target letters identified, and on speed: number of letters identified. Accuracy × Speed gives final score.

**Figure 2:**
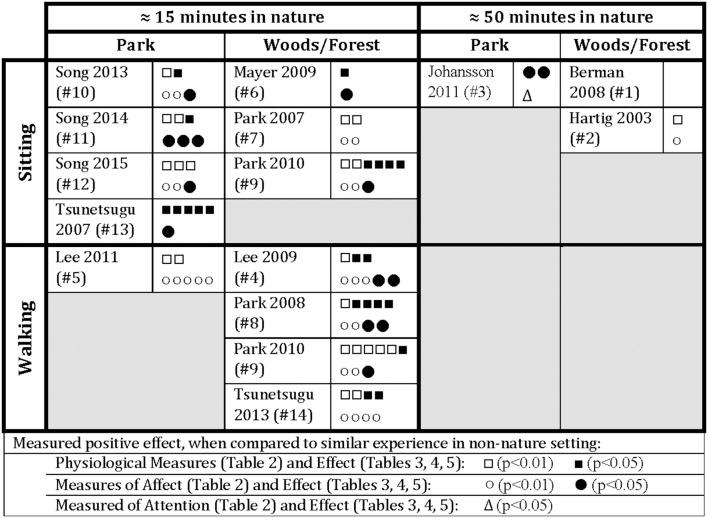
Reported effects or benefits of time in nature, as compared to similar experience in non-nature setting (summary of data from Tables 3–5).

Across all of the studies included in the review, it appears that time in nature does have a positive effect on physiology, affect, and attention (Tables 3–5). In sum, as presented in Figure 2 it appears that:

- *10–30 min of sitting outdoors* looking at or being in nature (Table 3) has the effect of decreasing biological and self-perceived markers of stress. When compared to those seated in an urban environment with a street view, often with traffic, those seated with a natural view (a campus green, a dense forest) showed stress reduction via **physiological measures:** significant decrease in heart rate (5 studies: #4, 5, 8, 9, 14), significant decrease in cortisol levels (3 studies: #4, 8, 9; a fourth study (#5) showed a non-significant decrease), significant decrease in blood pressure (3 studies: #4, 9, 14), significant increase in parasympathetic nervous system activity, and a significant decrease in sympathetic nervous system activity (4 studies: #5, 8, 9, 14, measured via heart rate variability); and via **psychological measures:** significant decrease in negative POMS scores and significant increase in positive POMS scores (3 studies: #5, 9, 14), significant decrease in STAI scores (1 study: #5), significant increase in SD scores (1 study: #5), significant decrease in SCL-90-R scores (1 study: #5), and significant increase in self-reported feelings of calm, comfort, being refreshed, and restored (3 studies: #4, 8, 14).
- *10–30 min of walking outdoors* in nature (Table 4) has the effect of decreasing markers of stress. When compared to those walking in an urban environment on concrete sidewalks alongside traffic, those walking in nature showed stress reduction via **physiological measures:** significant decrease in heart rate (5 studies: #9–13), significant decrease in cortisol levels (3 studies: #7, 9, 13), significant decrease in blood pressure (2 studies: #9, 13), significant increase in parasympathetic nervous system activity and a significant decrease in sympathetic nervous system activity (5 studies: #9–13, measured via heart rate variability), and a significant difference in neural/central nervous system activity (2 studies: #6, 7); and via **psychological measures:** significant decrease in negative POMS scores and significant increase in positive POMS scores (4 studies: #9–12), significant decrease in STAI scores (3 studies: #10–12), significant increase in SD scores (3 studies: #10–12), significant increase in PANAS scores (1 study: #6), and significant increase in self-reported feelings of calm, comfort, and refreshed (2 studies: #7, 13).
- *31–50 min of walking outdoors* in nature (Table 5) has the effect of decreasing markers of stress. When compared to those walking in an urban environment, on concrete sidewalks alongside traffic, those walking in nature showed stress reduction via **physiological measures:** significant decrease in blood pressure 30 min into a walk, although no difference at end (1 study: #2); and via **psychological measures:** significant increase in positive emotion ZIPERS scores and significant decrease in negative emotion ZIPERS scores (1 study: #2), significant increase in revitalization (EFI score, 1 study: #3), and significant decrease in feeling rushed (NMS score, 1 study: #3). There was no significant difference measured in perceived stress (PSS score, 1 study: #3), in overall happiness (OHS score, 1 study: #2), or in the positive subscales of the PANAS (1 study: #1). Those walking in nature also showed varying differences in **attention:** no significant difference via the NCPCT scale (1 study: #2), an increase in attention via the backwards digit-span task, and a significant decrease in attention via the SST.

**Table 3:** Measured effects of time in nature via included studies 10–30 min of sitting outdoors: natural vs. urban view.

**Dose**	**Study**	**Measured effect (from natural setting when compared to *urban setting*)**
15-min Dense forest view vs. *Urban street view*	#4 (Lee et al., 2009)	When compared to those sitting in the urban setting, those sitting in the forest showed: Physiology (cortisol, blood pressure, heart rate) - Lower cortisol levels before and after viewing (*p* < 0.01) - Lower diastolic blood pressure after viewing (*p* < 0.05) - Lower heart rate after viewing (*p* < 0.05) Positive feelings (self-report) - Higher measures for feelings of comfort after viewing (*p* < 0;01) and in the evening (*p* < 0.05) - Higher restorative effects after viewing (*p* < 0.01) - Higher feelings of refreshment after viewing (*p* < 0.05) and in the evening (*p* < 0.01)
15 min Campus park view vs. *Urban view*	#5 (Lee et al., 2011)	When compared to those sitting in the urban setting, those sitting in the forest showed: Physiology (heart rate, heart rate variability, cortisol) - A decrease in heart rate (*p* < 0.01) - An increase in parasympathetic HR variability (*p* < 0.01) - Lower cortisol levels after viewing. Psychological (SD) (POMS) (STAI) (SCL-90-R) - Higher values for three adjective pairs: comfortable-uncomfortable, soothed-aroused, and natural-artificial (*p* < 0.01) (SD) - Decreased measures for somatization, obsessive-compulsive, interpersonal-sensitivity, depression, anxiety, hostility, and paranoid ideation (*p* < 0.01) (SCL-90-R) - Lower levels of negative feelings: tension-anxiety, depression, anger-hostility, fatigue, and confusion (*p* < 0.01) (POMS) - Higher levels of vigor (*p* < 0.01) (POMS) - Decreased state of anxiety (*p* < 0.01) (STAI)
15 min Dense forest view vs. *Urban street view*	#8 (Park et al., 2008)	When compared to those sitting in the urban setting, those sitting in the forest showed: Physiology (cortisol, heart rate, heart rate variability) - Lower heart rate before and after viewing (*p* < 0.01) - Higher parasympathetic HR variability while viewing (*p* < 0.05) - Lower cortisol levels before viewing (*p* < 0.05), after viewing (*p* < .05), and the evening (*p* < 0.06) Positive feelings (self-reported) - Higher feelings of comfortability after viewing (*p* < 0.01) and in the evening (*p* < 0.05) - Higher feelings of calmness after viewing (*p* < 0.01) Self-perception - Higher refreshed feelings after viewing and in the evening (*p* < 0.05)
11–21 min Forest sit vs. *Urban sit*	#9 (Park et al., 2010)	When compared to those sitting in the urban setting, those sitting in the forest showed: Physiology (cortisol, heart rate, blood pressure, sympathetic nerve activity) - Lower cortisol levels (*p* < 0.01) - Lower heart rate (*p* < 0.01) - Lower systolic blood pressure (*p* < 0.01) - Lower diastolic blood pressure (*p* < 0.05) - Higher parasympathetic HR variability (*p* < 0.01) - Lower sympathetic HR variability (*p* < 0.01) Psychological (POMS) - A decrease in negative POMS subscales (Tension/Anxiety, Depression, Anger/Hostility, Fatigue, Confusion) (*p* < 0.01, *p* < 0.05) - An increase in the positive POMS subscale, Vigor (*p* < 0.01)
15 min Natural view vs. *Urban street view*	#14 (Tsunetsugu et al., 2013)	When compared to those sitting in the urban setting, those sitting in the forest showed: Physiology (blood pressure, heart rate) - Lower diastolic blood pressure (*p* < 0.05) - Higher parasympathetic HR variability (*p* < 0.01) - Suppression of sympathetic nervous activity (LF/HR ratio, *p* < 0.05) - Lower heart rate (*p* < 0.01) Psychological (POMS) - Lower Tension-Anxiety score (*p* < 0.01) - Lower Fatigue score (*p* < 0.01) - Lower Confusion score (*p* < 0.01) - Higher Vigor score (*p* < 0.01) Positive feelings (self-report) (refreshed Mackay et al., 1978) - Higher comfortability feelings (*p* = 0.00, *r* = 0.51) - Higher soothing feelings (*p* = 0.00, *r* = 0.53) - Higher natural feelings(*p* = 0.00, *r* = 0.59) - Higher refreshment feelings (*p* = 0.00, *r* = 0.55)

**Table 4:** Measured effects of time in nature via included studies 10–30 min of walking outdoors: natural vs. urban setting.

**Dose**	**Study**	**Measured effect (from natural setting when compared to *urban setting*)**
10 + 5 min Natural walk vs. *Urban walk*	#6 (Mayer et al., 2009)	When compared to those walking in the city, those walking in a natural area showed: Physiology (central nervous system) - A greater increase in CNS (*p* < 0.05) Psychological (PANAS) - A greater increase PANAS scores (*p* < 0.05)
20 min Forest area walk vs. *Urban walk*	#7 (Park et al., 2007)	When compared to those walking in the city, those walking in a forest area showed: Physiology (neural activity, cortisol) - A greater decrease in neural activity (*p* < 0.01) - A greater decrease in cortisol levels (*p* < 0.01) Self-perception (comfort, calm) - A greater increase in levels of calm (*p* < 0.01) - A greater increase in levels of comfort (*p* < 0.01)
11–21 min Forest walk vs. *Urban walk*	#9 (Park et al., 2010)	When compared to those walking in an urban environment, those walking in the forest showed: Physiology (cortisol, heart rate, blood pressure, sympathetic nerve activity) - Lower cortisol levels (*p* < 0.01) - Lower heart rate (*p* < 0.01) - Lower systolic blood pressure (*p* < 0.05) - Lower diastolic blood pressure (*p* < 0.05) - Higher parasympathetic HR variability (*p* < 0.05) - Lower sympathetic HR variability (*p* < 0.05) Psychological (POMS) - A decrease in negative POMS subscales (Tension/Anxiety, Depression, Anger/Hostility, Fatigue, Confusion) (*p* < 0.01, *p* < 0.05) - An increase in the positive POMS subscale, Vigor (*p* < 0.01)
15 min Urban park walk vs. *City area walk*	#10 (Song et al., 2013)	When compared to those walking in the city, those walking in the urban park showed: Physiology (heart rate, heart rate variability) - A greater decrease HR (*p* < 0.05) - A greater increase parasympathetic HR variability (*p* < 0.01) - A greater decrease sympathetic HR variability (*p* = 0.06) Psychological (SD) (POMS) (STAI) - A greater increase in SD score (*p* < 0.01) - A greater decrease in negative POMS scores and an increase in positive POMS scores (*p* < 0.01) - A greater decrease in STAI scores (*p* < 0.05)
15 min Urban park walk vs. *City street walk*	#11 (Song et al., 2014)	When compared to those walking in the city, those walking in the urban park showed: Physiology (heart rate, heart rate variability) - A greater decrease in HR (*p* < 0.05) - A greater increase in parasympathetic HR variability (*p* < 0.01) - A greater decrease in sympathetic HR variability (*p* < 0.01) Psychological (SD) (POMS) (STAI) - A greater decrease in STAI scores (*p* < 0.05) - A greater decrease in negative POMS scores, an increase in positive POMS scores (*p* < 0.05) - A greater increase in SD scores (*p* < 0.05)
15 min Urban park walk vs. *Urban street walk*	#12 (Song et al., 2015)	When compared to those walking in the city, those walking in the urban park showed: Physiology (heart rate, heart rate variability) - A greater decrease HR (*p* < 0.01) - A greater increase parasympathetic HR variability (*p* < 0.01) - A greater decrease sympathetic HR variability (*p* < 0.01) Psychological (POMS) (STAI) (SD) - A greater increase in SD score (*p* < 0.01) - A greater decrease in negative POMS scores and an increase in positive POMS scores (*p* < 0.05) - A greater decrease in STAI scores (*p* < 0.01)
15 +15 min Urban park walk vs. *Urban street walk*	#13 (Tsunetsugu et al., 2007)	When compared to those walking in the city, those walking in the urban park showed: Physiology (blood pressure, cortisol, heart rate, heart rate variability, immunoglobulin A) - A greater decrease in cortisol (*p* < 0.05) - A greater decrease in HR (*p* < 0.05) - A greater decrease in blood pressure (*p* < 0.05) - A greater increase in parasympathetic HR variability (*p* < 0.05) - A greater decrease in sympathetic HR variability (*p* < 0.05) Positive feelings (self-report) - An increase in feelings of “calm, comfortable, refreshed” (*p* < 0.05)

**Table 5:** Measured effects of time in nature via included studies 31–60 min of walking outdoors: natural vs. urban setting.

**Dose**	**Study**	**Measured effect (from natural setting when compared to *urban setting*)**
50-min Arboretum walk vs. *Urban walk*	#1 (Berman et al., 2008)	When compared to those walking in the city, those walking in the natural setting showed: Mood (PANAS—positive subscales) - No significant difference measured between groups Attention - A greater increase of 1.5 digits on backwards digit-span task (t(36) = 4.783, prep = 0.99)
50-min Nature reserve walk vs. *Urban city walk*	#2 (Hartig et al., 2003)	When compared to those walking in the city, those walking in the natural setting showed: Physiology (blood pressure) - A greater decrease in blood pressure, 30 minutes into walk (*p* < 0.01). Significance lost at the end of the trial. Emotion (ZIPERS)(OHS) - A greater increase in positive emotion, and a greater decrease in feelings of anger and aggressiveness (*p* < 0.01) (ZIPERS) - No significant difference measured in overall happiness (OHS) Attention (NCPCT) - No significant change in attention could be measured.
50-min Park walk vs. *Urban walk*	#3 (Johansson et al., 2011)	When compared to those walking in the city alone and with a friend, those in the natural setting showed: Affect (EFI scale) (NMS scale) - An increase in revitalization while walking alone and while walking with a friend. The greatest effect was observed while walking alone (*p* < 0.05) (EFI) - A decrease in feelings of being rushed while walking alone (*p* < 0.05) (NMS) Attention (Symbol Substitution Test) - A decline in attention score results (*p* < 0.05) < Result was unexpected> Perceived Stress (PSS) - No significant difference was noticed in PSS.

## Discussion

### Summary of Main Findings

The goals of this scoping review were to comprehensively scan the available literature to (1) identify what *accessible and sustainable dose of time in nature* appears to elicit a positive impact on *mental health* in people of college-age, (2) describe what types of engagement with nature (i.e., passive vs. active engagement) provide said impacts, (3) describe the most commonly used measures of effect, and (4) identify the strengths and gaps in the literature (methods and understanding) to guide future research. Strict search limits were used to screen more than 10,000 documents, ultimately identifying only 14 peer-reviewed manuscripts that met the scoping review inclusion criteria, where study subjects were of average college age (15–30 years old); where time in nature was explicitly measured in hours or minutes and limited to time that would be accessible to all college students in a sustainable, long-term way; and where the studies took place in an outdoor nature settings.

The 14 studies examined in this review revealed that as little as 10–20 min and up to 50 min of sitting or walking in a diverse array of natural settings has significant and positive impacts on key psychological and physiological markers, when contrasted with equal durations spent in urbanized settings.

To assess changes in the study participants' physiological and psychological markers of stress following time in nature, a total of 22 different measures were employed across the 14 studies, including biological measures of physiology, standardized and non-standardized measures of affect, and standardized and non-standardized measures of attention.

Within the included studies, statistically significant differences in physiological health markers of stress were associated with time in nature, including decreased heart rate, salivary cortisol, blood pressure, and sympathetic nervous system activity, and increased parasympathetic nervous system activity. Furthermore, within the included studies, statistically significant differences in psychological health markers of reduced stress were also attributed to time spent in nature, including decreased negative POMS scores (less anger-hostility, confusion-bewilderment, depression-dejection, fatigue-inertia, tension-anxiety); increased positive POMS scores (more vigor-activity): decreased STAI scores (lower anxiety); increased SD scores (greater sense of comfort, being soothed, feeling natural); increased PANAS scores (positive affect); and increased self-reported feelings of calm, comfort, and being refreshed.

The findings of this review are similar to studies of non-college-aged populations that have found evidence of stress reduction through engagement with nature (Fan et al., 2008; Benham et al., 2009; Hartig et al., 2014; Frumkin et al., 2017; Hansen et al., 2017; Antonelli et al., 2019; Hunter et al., 2019). On the strength of the papers included in this scoping review, there is no reason to doubt that these same stress-relieving benefits accrue to those of college age. This paper adds value to the literature in showing that a dose of as little as 10–20 min sitting or walking in an array of green spaces can have a meaningful impact in reducing stress, anger, anxiety, and in increasing vigor, comfort, positive affect, and a sense of feeling refreshed. Identifying and utilizing nature as an upstream easy, cost-effective tool to prevent and/or combat stress can help society alleviate a substantial health burden that contributes to and exacerbates myriad other negative physiological and psychological conditions (Maller et al., 2006).

### Limitations

Limitations of this scoping review include inclusion, heterogeneity, confounders, bias, and possibly scope. As noted above, this review only includes studies that were published and available/identifiable, and met the inclusion criteria. Although, a systematic and multi-pronged approach was used to identify eligible studies, it is possible that some were missed.

In reviewing across the 14 included studies, the researchers did note heterogeneity of study sites (countries and cities), meaning that findings may not be generalizable to all populations and locations. Three of the 14 studies were conducted in the United States, one in Sweden, and 10 in Japan. The Japanese studies were led by members of a seemingly related research team. Since the 1980s, the Japanese government has led a major initiative to connect its citizens with nature through “Shinrin-yoku,” or “forest bathing,” programs. Although there is no reason to doubt the rigor of these studies, a certain degree of scientific skepticism may be warranted. Finally, participant groups in more than half of the studies were male only, and it is unclear if the findings on male participants can be generalized to females in the same age group. Therefore, it would be appropriate to duplicate these studies in a multiplicity of locations, with equal cohorts of males and females.

This scoping review also summarized the noted benefits of time in nature, including physiological measures (such as heart rate, cortisol levels, and blood pressure), psychological measures (such as stress, happiness, calm, comfort, and restoration), and attention. While these are measures that prior research has shown to be affected by time in nature, these are also measures that may be affected by other confounding factors such as exercise (e.g., running, hiking). This scoping review does not take that into account. However, to limit confounding factors (knowing that exercise can impact these measures), and to support our goal of identifying accessible and sustainable doses of time in nature (activities that anyone could do, regardless of means), studies that included vigorous exercise, or explicitly focused on the effect of exercise in nature, were excluded in favor of studies that looked at accessible and routine activities, such as walking and sitting.

As a scoping review to inform future research or practice, risk of bias was not assessed within each of the included studies, as may be common in more formal systematic reviews. Of note, however, a recent systematic review led by Antonelli et al. (2019) did evaluate the quality of four studies included in this scoping review (Park et al., 2007, 2008, 2010; Tsunetsugu et al., 2007), rating them as as fair quality (NIH tool) and with high risk of bias (Cochrane tool). Related to this specific review, there may be bias in the summary of findings in that the authors have extracted these findings based on publications that were identified. Inherent bias may exist as it is possible that some studies on this topic were not published, especially those which may not have produced compelling or positive results. Although experts in the field were contacted to obtain the most up to date information, the authors acknowledge that not all relevant knowledge on this topic may have been captured.

Lastly, the purpose of this study was a scoping review, and as such, did not include a meta-analysis of results across studies. While a thematic analysis approach was used, based on within-study findings, interpretation and generalization of the results may be difficult due to the relatively small sample sizes for most included studies, and the variety of measures of effect used, including subjective measures.

### Conclusions

Stress, depression, and anxiety are affecting college students in the U.S. at alarmingly high rates, and there is an urgent need to find modalities for helping this population cope by providing individuals agency to improve their own mental health and well-being. An increasing number of studies have provided evidence that people who spend time in nature-rich environments benefit psychologically and physiologically. Although the heterogeneity of methodologies makes conclusions difficult, this scoping review summarizes studies that show that as little as 10–20 min of time spent sitting or walking in nature has a beneficial effect on college-aged adults' mental health, and that the same amount of time spent outdoors in urbanized settings does not have the same benefits.

Institutions of higher learning, as well as healthcare organizations and other entities that interact regularly with this population, should strongly consider providing access to nature on their campuses. They should also provide programs that encourage people in this age group to take advantage of this valuable resource. Several colleges and universities have instituted “Nature Rx” or “Park Prescription” programs that employ dedicated websites, health clinic-offered nature prescriptions, and nature experience classes to encourage all students, regardless of their health status, to spend time exploring the natural world (Razani et al., 2018; Rakow and Eells, 2019).

Researchers have begun to discuss access to nature in a public health and epidemiology context rather than simply as an issue of environmental psychology and design (Kuo, 2015; Frumkin et al., 2017; Fong et al., 2018). As an ever larger body of scientific evidence regarding the beneficial effects of time spent in nature is amassed, one can imagine this research having an impact on national health policies. Much as current policies provide guidelines for healthy diet and exercise, future strategies may stipulate the minimum time an individual should spend in nature each day or week.

Based on the limited number of published studies on this topic and with this population, more primary and translational research, implemented in a consistent manner, is warranted. To this end, the research team suggests six specific foci:

1. More applied research in this population in the U.S., based on campus settings, to evaluate the feasibility and impact of various types of interventions. This could include highly-controlled research using a cross-over design (differences in effect based on time in exposure 1 vs. exposure 2), and applied research studies that document and measure the effects of specific intervention types (i.e., park prescription, NatureRx, or course-based programs). In this area, papers on processes and outcomes—including successes and failures—would be of value.
2. More consistent use of similar measures and tools across studies to develop a larger pool of results that allow for meta-analyses. More focused adoption of a smaller number of measures and tools that could be readily used with larger numbers of study participants—with equal numbers of key demographics—could create a statistically stronger evidence base.
3. More collaboration across study sites to increase statistical power of studies, including larger sample sizes, consistency of measures, and consistency of similar activities across different regions and types of nature settings. Based on the studies summarized in this review, it appears that 10–20 min of time in nature has a positive effect on markers of mental health. Testing interventions, exposures (including frequency of repeated exposures), and benefits of college-age students across campuses and across seasons in the U.S. could provide some strong indications for public health interventions.
4. Deeper considerations for habitude or differences between groups, including those for whom time in nature is routine and the norm (vs. those who dislike nature), and those who live on a highly walkable campus (layout, climate, etc.) (vs. those who do not). Capturing data about routines and attitudes as pre-study may help control for varying measured effects.
5. Deeper considerations of the social aspects of nature experiences, and the complementary benefits that socialization brings. For example, is it more beneficial to engage in solitary or group nature-based experiences? Researchers might also measure the impact of different types of nature settings, such as, does a walk through an undisturbed woodland provide greater benefit than through a park-like setting?
6. Potential use of a public health approach for research, using big data, longitudinal analysis, and interactional analysis, such as the interaction between time spent in nature and other physical activity, or with body mass index. Successful research teams will include an interdisciplinary mix of scholars in design (architecture, landscape architecture, and planning), psychology, epidemiology, public health, pharmacology, and medicine.

## Author Contributions

GM, DR, and EE co-developed the concept of the paper. EE led the scoping review process. GM acted as the primary convener of the author group. GM, DR, and CM reviewed and adjudicated each identified paper and outlined the analysis. GM, CM, and SS conducted the analysis. DR, NS, and GM co-outlined the paper. DR, GM, EE, and NS co-wrote the paper. GM acted as the primary editor of the paper.

### Conflict of Interest

The authors declare that the research was conducted in the absence of any commercial or financial relationships that could be construed as a potential conflict of interest.

## Footnotes

^1^The Cambridge Dictionary Online. Available online at: https://dictionary.cambridge.org/us/dictionary/english/nature

^2^The Necker Cube Test. Available online at: http://new.censusatschool.org.nz/wp-content/uploads/2013/09/NECKER-CUBE-FINAL.docx
